# Enhanced Microsphere-Assisted Picosecond Laser Processing for Nanohole Fabrication on Silicon via Thin Gold Coating

**DOI:** 10.3390/mi12060611

**Published:** 2021-05-26

**Authors:** Qiuling Wen, Xinyu Wei, Pengcheng Zhang, Jing Lu, Feng Jiang, Xizhao Lu

**Affiliations:** 1 Institute of Manufacturing Engineering, Huaqiao University, Xiamen 361021, China; weixinyu163@126.com (X.W.); pc0604@foxmail.com (P.Z.); lujing26@hqu.edu.cn (J.L.); jiangfeng@hqu.edu.cn (F.J.); 2 MOE Engineering Research Center for Brittle Materials Machining, Institute of Manufacturing Engineering, Huaqiao University, Xiamen 361021, China; 3 College of Mechanical Engineering and Automation, Huaqiao University, Xiamen 361021, China; luxizhao@hqu.edu.cn

**Keywords:** picosecond laser, silicon substrate, gold film, nanohole arrays, polystyrene microspheres

## Abstract

The nanohole arrays on the silicon substrate can effectively enhance the light absorption in thin film silicon solar cells. In order to optimize the solar energy absorption, polystyrene microspheres with diameters of 1 μm are used to assist picosecond laser with a wavelength of 1064 nm to fabricate nanohole arrays on silicon substrate. The experimental results show that the morphology and size of the silicon nanoholes strongly depend on the laser fluence. At 1.19–1.59 J/cm^2^ laser fluences, well-ordered arrays of nanoholes were fabricated on silicon substrate, with diameters domain from 250 to 549 nm and depths ranging from 60 to 99 nm. However, large amounts of sputtered nanoparticles appeared around the silicon nanoholes. To improve the surface morphology of silicon nanoholes, a nanolayered gold coating is applied on silicon surface to assist laser processing. The results show that, for gold-coated silicon substrate, sputtered nanoparticles around the nanoholes are almost invisible and the cross-sectional profiles of the nanoholes are smoother. Moreover, the ablation rate of the nanoholes on the gold-coated silicon substrate have increased compared to that of the nanoholes on the uncoated one. This simple method allows fast fabrication of well-ordered nanoholes on silicon substrate without sputtered nanoparticles and with smooth inner surface.

## 1. Introduction

Fabrication of nanohole arrays on the silicon substrate can effectively suppress light reflection, thereby increasing the light absorption of the silicon substrate [1,2,3,4]. Therefore, the nanohole arrays on silicon substrate can improve the light absorption of silicon-based solar cells [5]. There are many ways to process nanohole arrays, including nanoimprint lithography [6,7], focused ion beam processing [8], electron beam processing [9], electrochemical processing [10] and laser processing [11], etc. Laser processing is a fast and simple method for drilling holes on silicon substrate [12,13], but the diffraction limit limits the minimum feature size in laser surface patterning technologies, such as lithography and laser direct writing [14]. Microspheres provide a mechanism for manipulating light in ways that traditional optical components cannot achieve, thus overcoming the diffraction limit of light [15,16]. Since each microsphere acts as a micro-lens, thus it can focus light into nanometer scale. Moreover, the contact region between the microspheres and the substrate has a near-field enhancement effect, which facilitates laser to remove material [17,18,19,20]. Therefore, microsphere-assisted laser processing has been widely used to fabricate nanohole arrays on various substrates. Zhou et al. [21] used silicon microspheres with a diameter of 1 μm to assist femtosecond laser directly pattern nanoholes on a glass substrate. The diameter of the nanoholes is in the range of 200–300 nm. Miyanishi et al. [22] studied the preparation of nanoholes via gold nanoparticles assisted femtosecond laser at different incident angles. Tanaka et al. [23] found that the use of high dielectric constant TiO_2_ particles assisted femtosecond laser can fabricate nanohole patterns on low refractive index SiO_2_ and high refractive index Si substrates. Hence, microsphere-assisted laser processing is a simple and fast method to fabricate nanohole arrays. However, many undesired sputtered nanoparticles are observed on the outer surface of silicon nanoholes by microsphere-assisted laser direct processing. In this paper, to improve the surface morphology of the nanoholes, a nanolayered gold coating was deposited on the silicon surface and this has not yet been explored. The result showed that the undesired nanoparticles around the nanoholes were significantly reduced. In addition, with the help of gold nanolayer, the internal morphology of silicon nanoholes was improved and the ablation rate was increased as well.

## 2. Experimental Methods

**Figure 1:**
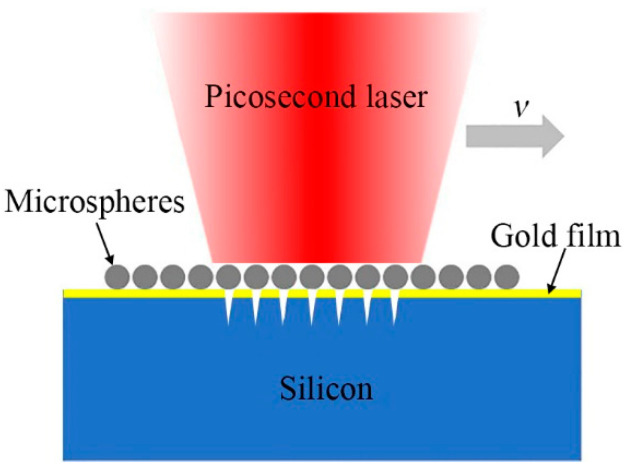
Schematic diagram of microsphere-assisted picosecond laser processing nanohole arrays on silicon substrate.

A commercial laser device with a central wavelength of λ = 1064 nm was used (BGL-1064-30B, BWT Laser Ltd., Tianjin, China), which generated 15 picosecond laser pulses. The repetition rate of the laser is 200 kHz. The beam quality factor M^2^ is smaller than 1.4. The laser polarization direction is perpendicular to the laser scanning direction. The laser beam was focused on the sample surface to a focal spot diameter of 80 μm (1/e^2^ intensity). The maximum laser fluence was limited to 7.16 J/cm^2^. The specimens used in the experiment are <100> oriented silicon substrates (Hefei Kejing Materials Technology Co., Ltd., Hefei, China) with dimensions of 10 × 10 × 0.5 mm^3^ (length × width × height), which were polished on top surface with surface roughness (Sa) <5 Å. A self-assembled monolayer of 1-μm-diameter polystyrene microspheres was applied on the silicon surface in order to assist picosecond laser to fabricate nanohole arrays. To improve the surface morphology of silicon nanoholes, a 10 nm-thick gold film was deposited on the silicon substrate. The specimen was mounted onto a micro-processing platform, and the picosecond laser was focused on the specimen surface. As shown in Figure 1, the microspheres within the irradiated area focused the incident laser beam into nanometer scale, and nanoholes were generated on silicon substrate. Moreover, large area nanoholes can be obtained by laser beam scanning. And the scanning speed (*v*) of the laser is 6000 mm/s.

The surface morphologies of nanoholes were evaluated with scanning electron microscopy (Apreo, FEI, Hillsboro, OR, USA). The three-dimensional (3D) morphologies and dimensions of nanoholes were characterized with atomic force microscopy (Dimension Icon, Bruker, Tip: ScanAsyst Air, silicon tip on nitride lever, Karlsruhe, Germany). All experiments were carried out in the atmosphere and room temperature (25 °C).

The monolayer of hexagonally arranged polystyrene microspheres was obtained by the following method. First, pour a proper amount of deionized water into the beaker, next use a pipette to suck 30 μL polystyrene microsphere mixed solution (add anhydrous ethanol and deionized water), then inject the microspheres solution to the water surface of the beaker slowly. After that add sodium dodecyl sulfonate (SDS) solution to the microspheres solution, which is put into the water bath in advance and heated at constant temperature. The SDS solution can effectively change the water surface tension, which makes the arrangement of microspheres more compact. When the volume ratio of water to ethanol is 1:1, the concentration of SDS solution is 6 wt%, and the volume of SDS solution is 1 mL, a monolayer of hexagonally arranged microspheres can be obtained on the solution surface.

**Figure 2:**
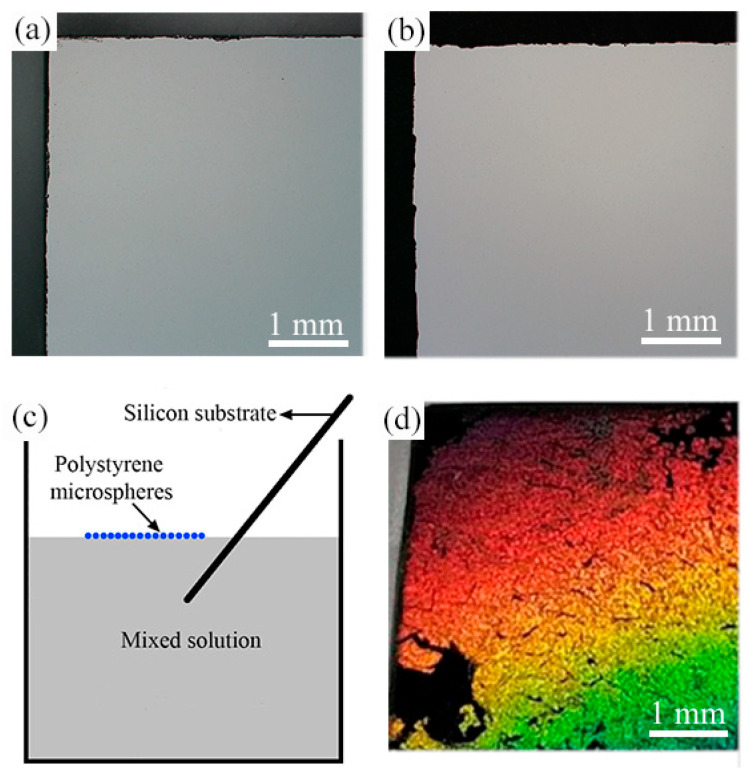
Optical microscope images of (**a**) uncoated silicon substrate and (**b**) gold-coated silicon substrate. (**c**) Schematic diagram of transferring a monolayer of polystyrene microspheres on solution surface to the silicon substrate. (**d**) Optical image of the monolayer of polystyrene microspheres on the gold-coated silicon surface.

**Figure 3:**
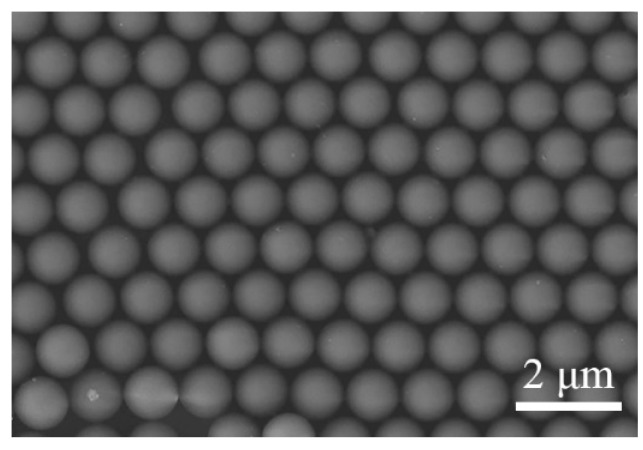
Scanning electron microscopy (SEM) image of a monolayer of hexagonally arranged polystyrene microspheres on the silicon surface.

The contact angle of the silicon substrate is 95.7°, which was measured by contact angle measuring instrument (JC2000D, Powereach, Shanghai, China). Therefore, the silicon substrate is hydrophobic, which is not conducive to the adsorption of microspheres on the silicon surface. In order to improve the hydrophilicity of the silicon substrate, the silicon substrate was placed in the piranha solution to stand for 24 h. After the hydrophilic treatment, the contact angle of the silicon substrate was reduced to 15.5°. Monolayer densely-arranged polystyrene microspheres on solution surface were transferred to the silicon surface using the method given in [24]. The detailed steps of the transfer method are described as follows. First, dip the silicon substrate without gold film (Figure 2a) and with gold film (Figure 2b) into the beaker at a certain angle, then slowly move the silicon substrate below the polystyrene microspheres (Figure 2c), and slowly lift the silicon substrate, so that the mono-dispersed polystyrene microspheres can be transferred onto the silicon surface. Finally, put the silicon substrate covered with polystyrene microspheres into the oven for drying, and a hexagonal monolayer of polystyrene microspheres on the silicon surface can be obtained. The optical microscope image of the monolayer of polystyrene microspheres on the gold-coated silicon surface is shown in Figure 2d. The SEM image of a hexagonal monolayer of polystyrene microspheres on the silicon surface is shown in Figure 3.

## 3. Results and Discussion

### 3.1. Influence of Gold Film on the Surface Morphology of Nanohole Arrays

The morphology and size of nanoholes depend strongly on the laser fluence. The experiment results show that, when the laser fluence is lower than 1.19 J/cm^2^, no nanoholes are generated on the surface of the silicon substrate. However, when the laser fluence is above 1.99 J/cm^2^, the polystyrene microspheres on the silicon substrate are directly burned and no nanoholes are generated. Therefore, in the experiment, the laser fluence was set from 1.19 to 1.79 J/cm^2^ in 0.2 J/cm^2^ intervals.

**Figure 4:**
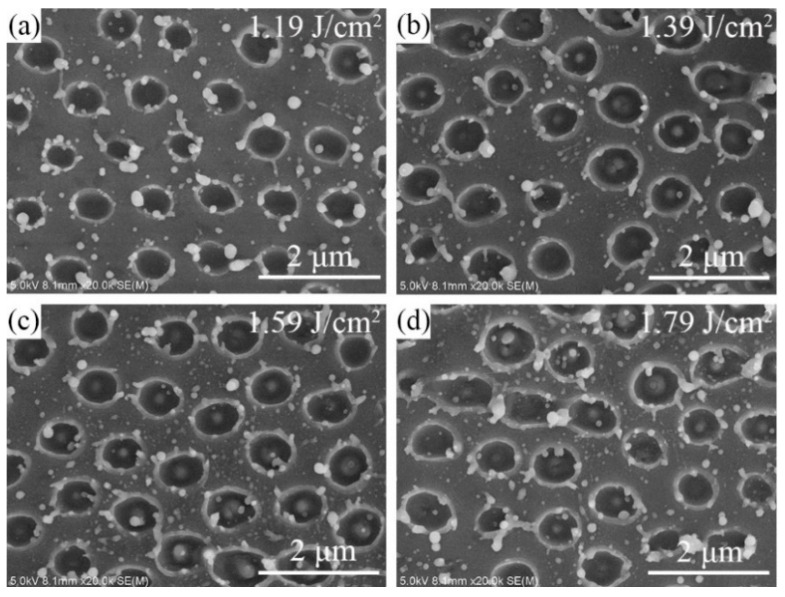
SEM images of nanohole arrays on uncoated silicon substrate fabricated by picosecond laser with (**a**) 1.19, (**b**) 1.39, (**c**) 1.59, (**d**) 1.79 J/cm^2^ laser fluences, respectively.

Figure 4a–d shows scanning electron microscopy (SEM) images of the nanohole arrays on silicon substrate fabricated by picosecond laser with 1.19, 1.39, 1.59, 1.79 J/cm^2^ laser fluences, respectively. One can see that there are no polystyrene microspheres on the silicon substrate after laser irradiation. This is because the microspheres are easily removed by ablation due to the low ablation threshold of polystyrene [25]. Furthermore, many undesired particles with diameters of 50–200 nm have emerged around the nanoholes, and pronounced molten materials are observed at the edge of the nanoholes. At laser fluences of 1.19–1.59 J/cm^2^, the shape of the nanoholes is circular. However, as laser fluence further increases to 1.79 J/cm^2^, some nanoholes become larger with an ellipse shape and stick together, as shown in Figure 4d. Moreover, as the laser pulse energy is larger than 1.39 J/cm^2^, nano-bumps develop in the center of the nanoholes (see Figure 4b–d).

The formation mechanism of nano-bumps in the silicon nanoholes was that surface melting led to the excitation of the convective fluxes within the melting layer. The temperature gradients in the transverse and longitudinal direction are produced in the molten pool under laser irradiation. The direction of the temperature gradients has a direct impact on the direction of the surface tension, molten material flows from the edge of the hole to the center, and a nano-bump is generated in the center of the nanohole [14,26].

**Figure 5:**
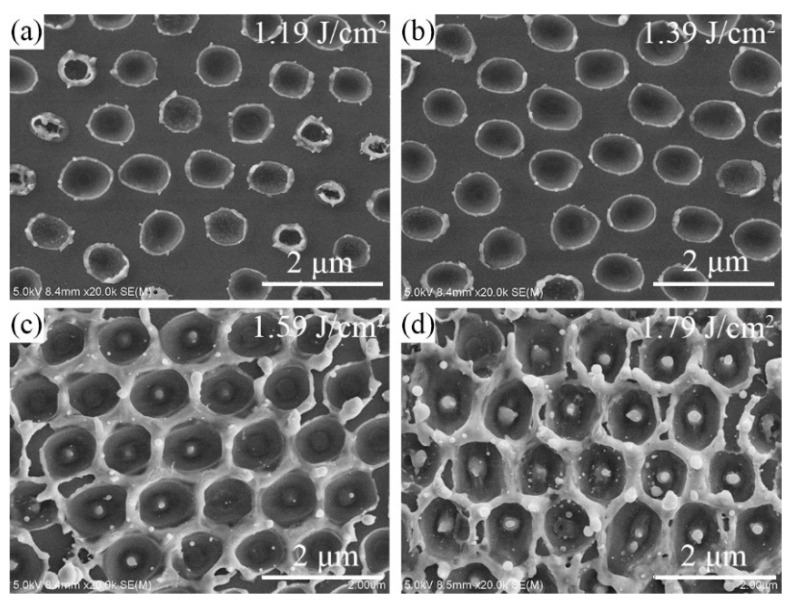
SEM images of nanohole arrays on gold-coated silicon substrate which were fabricated by picosecond laser with (**a**) 1.19, (**b**) 1.39, (**c**) 1.59, (**d**) 1.79 J/cm^2^ laser fluences, respectively.

In order to reduce the undesired particles around the nanoholes, a 10 nm-thick gold coating was deposited on silicon substrate to facilitate laser fabrication of nanoholes. As shown in Figure 5a,b, sputtered nanoparticles are not observed around the nanoholes on the gold-coated silicon substrate compared with those on uncoated one. Although some molten materials have remained at the edge of each nanohole, the edges of all nanoholes are relatively clean and clear. The reason for the improvement of surface morphology of nanoholes is as follows. The gold film increases the laser energy absorption of the silicon substrate, which allows the silicon material to reach the vaporization temperature quickly. As the laser fluence increases to 1.59 and 1.79 J/cm^2^, the diameters of nanoholes enlarge, and the gaps of nanoholes reduce, causing these holes to be tightly connected together and arranged closely in a hexagonal array, see Figure 5c,d. Moreover, a thick layer of molten material appears on the edges of nanoholes and a few small nanoparticles are observed within the nanoholes. In addition, silicon nano-bumps emerge in the center of nanoholes at 1.59 J/cm^2^ laser fluence, and they grow bigger and higher as the laser fluence increased to 1.79 J/cm^2^.

### 3.2. Influence of Gold Film on the Size and Internal Morphology of Nanohole Arrays

**Figure 6:**
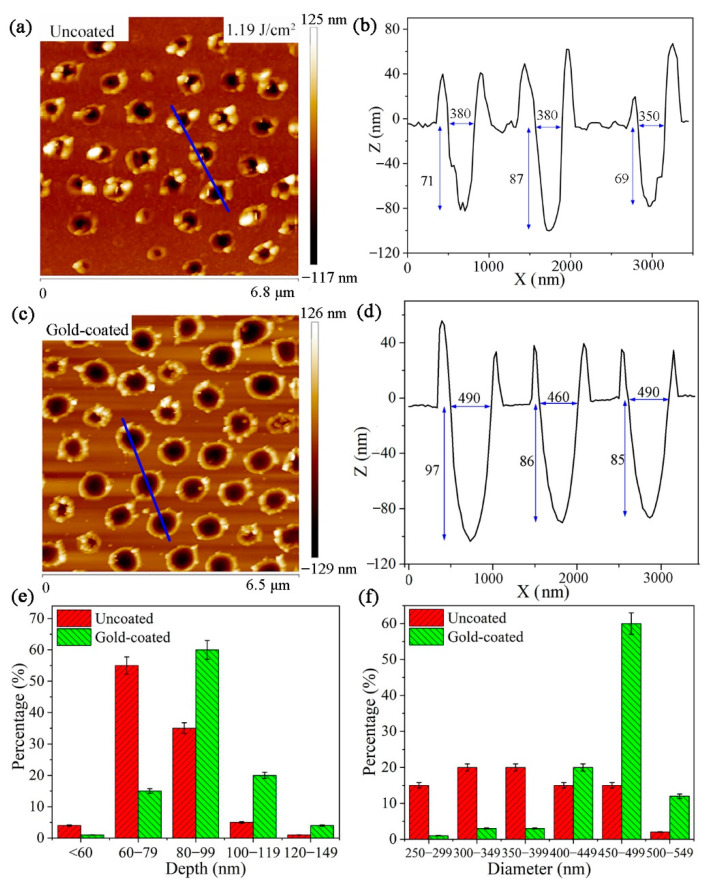
(**a**,**c**) Atomic force microscopy (AFM) images of the nanoholes on uncoated and gold-coated silicon substrate produced with 1.19 J/cm^2^ laser fluence, respectively. (**b**,**d**) The cross-sectional profile of the selected nanoholes in (**a**,**c**), respectively. (**e**,**f**) Histograms of the depths and diameters of the nanoholes in (**a**,**c**), respectively.

In order to more intuitively show the internal morphology and size of the nanoholes, Atomic force microscopy (AFM) was used to evaluate the internal morphology and size of nanoholes created on silicon substrate coated with and without gold film. Figure 6a,c shows the AFM morphology of nanohole arrays produced with 1.19 J/cm^2^ laser fluence for uncoated and gold-coated silicon substrate, respectively. The cross-sectional profiles of the three selected nanoholes in Figure 6a,c are depicted in Figure 6b,d, respectively. In Figure 6b, the cross-sectional profile of the nanoholes on the uncoated silicon substrate is not smooth. From Figure 6d, one can see that the cross-sectional profile of the nanoholes on the gold-coated silicon substrate presents a smooth parabolic curve. This means that the gold nanolayer makes the inner surface of the silicon nanoholes smoother.

The diameters and depths of nanoholes can be get from the cross-sectional profiles of the nanoholes which were measured by AFM. As seen in Figure 6b, the depths of the three selected nanoholes on uncoated silicon substrate are 71, 87, and 69 nm, respectively, and their diameters are 380, 380, and 350 nm, respectively. While the depths of the three selected nanoholes on gold-coated silicon substrate are 97, 86, and 85 nm, respectively, and the corresponding diameters are 490, 460, and 490 nm, respectively. We further made a statistical analysis of the diameters and depths of the nanoholes within the measurement area of AFM. Figure 6e,f show the depths and diameters distributions of all the nanoholes in Figure 6a,c, respectively. It can be seen that, for nanoholes on uncoated silicon substrate, the depths of nanoholes are mainly distributed from 60–79 nm, and their diameters are distributed in the range of 250–499 nm. For nanoholes on gold-coated silicon substrate, the depths and diameters of the nanoholes are mainly distributed in domain from 80–99 nm and 450–499 nm, respectively. This indicates that the gold film can also increase the depths and diameters of the nanoholes compared to those of nanoholes on the uncoated one.

**Figure 7:**
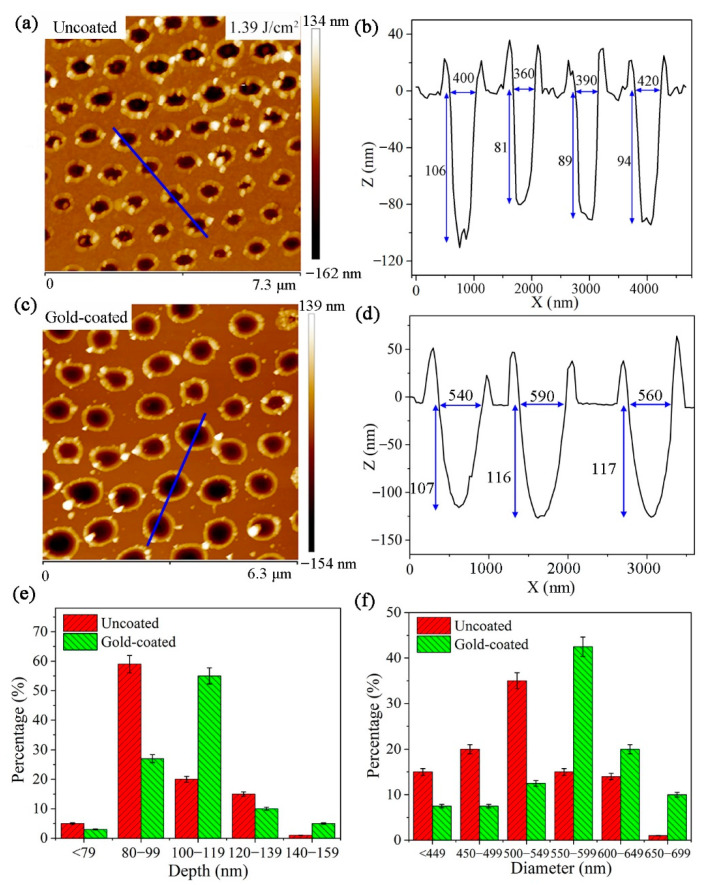
(**a**,**c**) AFM images of the nanoholes on uncoated and gold-coated silicon substrate produced with 1.39 J/cm^2^ laser fluence, respectively. (**b**,**d**) The cross-sectional profile of the selected nanoholes in (**a**,**c**), respectively. (**e**,**f**) Histograms of the depths and diameters of the nanoholes in (**a**,**c**), respectively.

As the laser fluence increased to 1.39 J/cm^2^, the AFM image of the nanohole arrays on the uncoated and gold-coated silicon substrate are shown in Figure 7a,c, respectively. Figure 7b depicts the cross-sectional profile of the selected nanoholes in Figure 7a. The corresponding depths of nanoholes are 106, 81, 89 and 94 nm, respectively, and their diameters are 400, 360, 390 and 420 nm, respectively. Figure 7d shows the depths of the selected nanoholes in Figure 7c are 107, 116, and 117 nm, respectively, and their diameters are 540, 590, and 560 nm, respectively. As seen in Figure 7e, the depths of the nanoholes on uncoated and gold-coated silicon substrate are mainly distributed in the range of 80–99 nm and 100–119 nm, respectively. Figure 7f displays the diameters of the nanoholes are distributed in domain from 500–549 nm for uncoated silicon substrate and 550–599 nm for gold-coated one. This also indicates that the depths and diameters of the nanoholes have increased by depositing a gold nanolayer on silicon surface.

**Figure 8:**
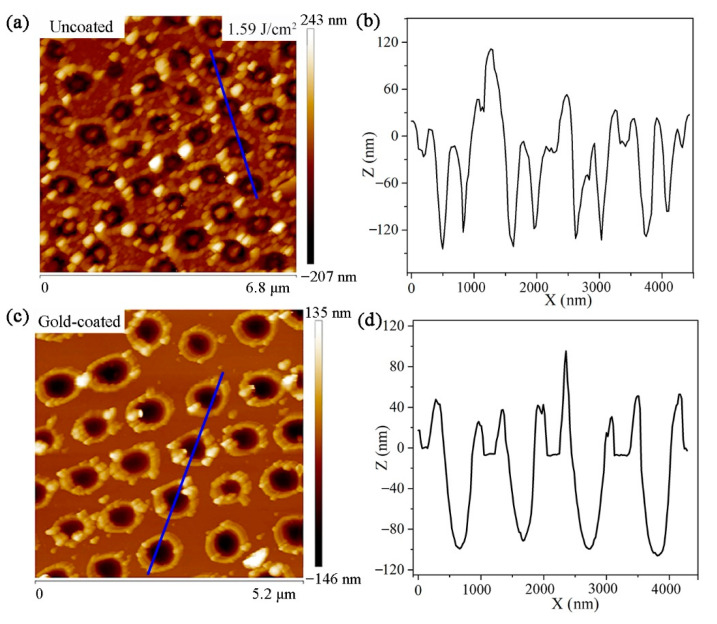
(**a**,**c**) AFM images of the nanoholes on uncoated and gold-coated silicon substrate produced with 1.59 J/cm^2^ laser fluence, respectively. (**b**,**d**) The cross-sectional profile of the selected nanoholes in (**a**,**c**), respectively.

As shown in Figure 8a, with further increasing laser fluence to 1.59 J/cm^2^, the ablation of silicon substrate is more intense, and there are many sputtered nanoparticles appeared around the nanoholes. As shown in Figure 8b, it can be seen that there are nano-bumps in the center of the nanoholes on the uncoated silicon substrate. However, as shown in Figure 8c, the sputtered particles around the nanoholes are significantly reduced for gold-coated silicon substrate. From Figure 8d, one can see that no nano-bumps are formed in the center of the nanoholes on the gold-coated silicon substrate.

**Figure 9:**
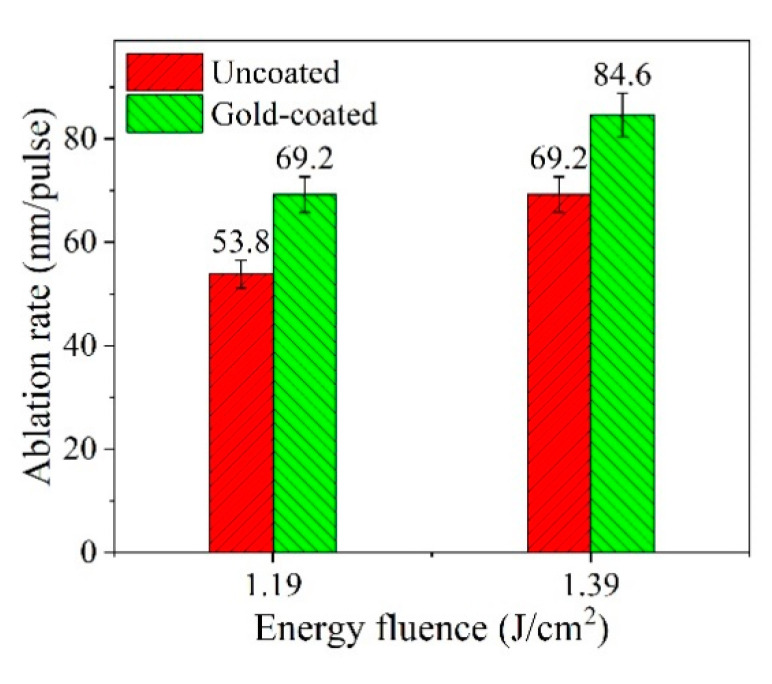
The ablation rate of nanoholes on uncoated and gold-coated silicon substrate.

The ablation rate of nanoholes was calculated by ablation depth per pulse. As shown in Figure 9, at 1.19 J/cm^2^ laser fluence, the ablation rate of the nanoholes is 53.8 nm/pulse for the uncoated silicon substrate and 69.2 nm/pulse for the gold-coated one. This indicates that the ablation rate of nanoholes on gold-coated silicon substrate was increased by 28.6% compared with that of nanoholes on uncoated one. As the laser fluence was increased further to 1.39 J/cm^2^, the ablation rate of nanoholes on gold-coated silicon substrate is 84.6 nm/pulse, which increases by 22.3% compared with that (69.2 nm/pulse) of nanoholes on the uncoated one.

The influence of the gold layer on the fabrication of nanoholes is described as follows. The gold film on the silicon substrate can provide a large amount of free electrons. These free electrons can enhance the energy coupling efficiency between incident laser beam and silicon substrate. Furthermore, the lattice temperature of the gold-coated silicon substrate within the laser irradiation region increases faster and higher than that of the uncoated one, which causes the silicon material to be removed more quickly by vaporization [27]. Thus, the ablation rate of the gold-coated silicon substrate is higher than that of the uncoated one, and fewer nanoparticles were deposited on the surface of the gold-coated silicon substrate due to the vaporization of silicon material. In addition, the hot electrons in the gold film moves randomly after absorbing the energy of photons. This allows the laser energy to be uniformly deposited on the surface of the silicon substrate [28,29]. Therefore, the inner morphologies of silicon nanoholes become better.

## 4. Conclusions

In summary, we mainly studied the microsphere-assisted laser fabricating nanohole arrays on silicon substrate coated with and without gold film. In order to optimize the light energy absorption of silicon solar cells, polystyrene microspheres with diameters of 1 μm and a laser wavelength of 1064 nm were chosen in the experiment. It was found that the laser fluence has significant impacts on the morphology and size of the nanoholes. The experimental results show that many sputtered nanoparticles appeared around the nanoholes on silicon substrate. After a gold nanolayer is applied on the silicon surface, almost no sputtered particles are observed on the outer surface of silicon nanoholes and the cross-sectional profiles of the nanoholes become smoother. This implies that the gold nanolayer can strongly improve the surface morphology and internal morphology of the silicon nanoholes. At 1.19 and 1.39 J/cm^2^ laser fluence, well-ordered nanohole arrays without sputtered particles can be obtained on gold-coated silicon substrate. The corresponding diameters of the nanoholes are distributed in 450–599 nm, and their depths are mainly distributed in the range of 80–119 nm. Moreover, the ablation rate of nanoholes at 1.39 J/cm^2^ laser fluence increases from 69.2 to 84.6 nm/pulse by depositing a 10 nm-thick gold film on silicon surface. This work provides technical support for the processing of nanohole arrays. The nanohole array textured silicon surface is believed to be promising for light-absorption enhancement in photovoltaic devices.

## Data Availability

Not applicable.
